# THE IMPACT OF CULTURE CHANGE ON FINANCIAL PERFORMANCE OF HIGH MEDICAID NURSING HOMES

**DOI:** 10.1093/geroni/igz038.560

**Published:** 2019-11-08

**Authors:** Justin C Lord, Ganisher K Davlyatov, Akbar Ghiasi, Robert Weech-Maldonado

**Affiliations:** 1 Louisiana State University at Shreveport, Shreveport, United States; 2 University of Alabama at Birmingham, Birmingham, Alabama, United States

## Abstract

This study examines the association between culture change artifacts and financial performance among under-resourced nursing homes (70% or higher Medicaid census). Culture change represents a transformational process to become person-centered, through staff and resident empowerment. Cultural artifacts represent the physical evidences that culture change is occurring. In this study, we focus on the workplace (nurse staffing consistent assignments) and leadership (residents engagement) artifacts to assess the relationship between culture change practices and performance. Survey data came from 387 nursing home directors from 2016- 2018, merged with secondary data from LTCFocus, Area Health Resource File, and Medicare Cost Reports. The dependent variable consisted of the total profit margin (%), while the independent variables comprised composite scores for leadership (0-25) and workplace artifacts (0-15). Control variables included organizational-level (ownership, chain affiliation, size, occupancy rate, and Medicare and Medicaid payer mix), and county-level factors (Medicare Advantage penetration, per capita income, educational level, unemployment rate, poverty level and competition). Multivariate regression was used to model the relationship between cultural change artifacts and financial performance. Workplace artifacts in nursing homes were found to be associated with significantly higher profit margin (β = 0.30, p < 0.05), while leadership artifacts were not. Culture change practices aimed at improving nursing staff consistent assignments are associated with better financial performance. Given increasing nursing home market competition and declining resources for high Medicaid nursing homes, facilities with a greater emphasis on workplace culture may be able to perform better financially among these under-resourced facilities.

